# Effect of parental attitudes on the practice and medicalization of female genital mutilation: a secondary analysis of Egypt Health Issues Survey, 2015

**DOI:** 10.1186/s12905-022-01834-7

**Published:** 2022-06-27

**Authors:** Mirette Aziz, Omaima Elgibaly, Fatma Elzahraa Ibrahim

**Affiliations:** 1 Department of Public Health & Community Medicine, Assiut University, Asyût, Egypt; 2 Assiut University Students Hospital, Asyût, Egypt

**Keywords:** Female genital mutilation, Medicalization, Egypt, Fathers

## Abstract

**Background:**

Despite the observed decrease in female genital mutilation (FGM) prevalence, it is increasingly being medicalized. We examined the attitudes of both parents towards the FGM practice in Egypt, and highlighted the effect of fathers’ decision making and attitudes towards FGM and violence on FGM practice and medicalization.

**Methods:**

This study is a secondary analysis of Egypt Health Issues Survey (EHIS), 2015. The 2015 EHIS involved a systematic random selection of a subsample of 614 Shiakhas/villages out of the 884 shiakhas/villages that had been chosen as Primary Sampling Units in the 2014 EDHS. Descriptive statistics of the study sample and parents’ attitudes was performed. Three indices were created describing; mothers’ and fathers’ attitudes towards FGM, decision making and rejecting violence against women. Bivariate and multivariable analyses were conducted to identify predictors of FGM practice and medicalization.

**Results:**

A considerable proportion of EHIS girls; 16.4% were circumcised and 36% of girls were expected to be circumcised. More than two thirds of circumcised girls were circumcised by a physician; namely 67% and 13.5% by nurses. The majority of mothers (88.4%) and fathers (84.8%) believed that FGM practice should continue. They believed that FGM is a religious obligation (72.3% of mothers and 73% of fathers). Parents believed that husbands prefer a circumcised wife (81.1% and 82.5% of mothers and fathers respectively). Being in the poorest wealth quintile (OR = 4.2, p < 0.001) and living in rural Upper Egypt (OR = 4.55, p < 0.001) were the predictors of FGM practice, while medicalization was more likely among the rich and educated parents. Parents’ attitudes supporting FGM was significantly associated with its practice (OR = 1.97, p < 0.001, for mothers and OR = 1.27, p < 0.001, for fathers). Rejecting violence against women was associated with less likelihood of practicing FGM (OR = 0.89, p < 0.05) and more likelihood of its medicalization (OR = 1.25, p < 0.01).

**Conclusion:**

More attention should be paid to enforce the laws against FGM practice by health care providers. Raising the community awareness on girls’ human rights and elimination of FGM practice which is a severe form of violence against women and gender inequality in sexual rights should be prioritized with targeting men in FGM programs.

**Supplementary Information:**

The online version contains supplementary material available at 10.1186/s12905-022-01834-7.

## Introduction

Although Female Genital Mutilation (FGM) is internationally considered a harmful practice, it is increasingly being medicalized [1]. Despite of the global efforts to eradicate FGM, every year, three million girls are subjected to this harmful practice mostly in Africa and Asia [2]. FGM violates the rights to health, security and physical integrity of girls and women and violates the right to life when the procedure results in death [1, 3, 4].

Practicing and justifying FGM reflect the cultural and social dimensions of the community where it is practiced. The cultural ideals of beauty and cleanliness of the circumcised genital organs is deeply entrenched in some communities [5, 6]. Gender inequality norms also urges the practice of FGM to control women’s sexuality and safeguard the honor of the family by ensuring virginity among young girls and marital fidelity among married women [7]. Moreover, in some conservative communities, FGM would confer girls the status of eligibility for marriage [7, 8]. Attitudes toward FGM are influenced by individuals’ social environment, which tends to pressure and socialize people toward conformity. The extent to which individuals and families can withstand such pressures depends on the available sources of status, as well as on their exposure to other social environments and influences. Therefore, not all groups are equally likely to change their attitudes toward FGM [9].

A decrease in the FGM prevalence in Egypt has been observed in the ages 15–17 from 74% in 2008 to 61% in 2014. Looking also at the differences in the percentages expected to be circumcised by the daughter’s age, it appears that there will be a steady decline in the proportion of young women who will be circumcised in Egypt, based on mothers’ stated intentions [10]**.**

However, rates of FGM medicalization in Egypt are more extensive than in any other country where FGM is practiced, and has increased from 55% in 1995 to 74% in 2014 [10, 11]. Favoring FGM by a medical professional is attributed to the community perception that it would minimize pain and other adverse consequences, such as bleeding and infection, while sustaining the practice to meet the cultural demand and satisfying the community expectations [1, 12].

Females, in a patriarchal society, are unable to leave their daughters uncircumcised, even if they want to, without the active support of their social network, especially fathers [13, 14]. The participation of fathers in the decision-making of not performing FGM may free the mothers from the community social pressure and responsibility of carrying on traditions and create a more favorable environment to stop FGM practice [13].

Despite the importance of fathers’ attitudes in the decision making of performing FGM, fathers’ role is usually overlooked and the existing research discussing the role of men in the decision making process of FGM is very limited. As we believe in the role of fathers in the decision making process of FGM, we thought to perform a secondary analysis of Egypt Health issues survey (EHIS) data [15], which is the last representative national survey conducted in Egypt. This study aimed to describe the attitudes of parents of circumcised girls towards FGM and to identify predictors of practicing FGM and FGM medicalization.

## Methods

### Type of the study

The study is a secondary analysis of data from Egypt Health Issues Survey (EHIS), 2015.

### Description of EHIS, 2015

The EHIS survey contains data on large nationally representative samples of all women, not only ever married women as Egypt Demographic Health Survey, men and children. The sample of EHIS has 27,549 eligible persons; 9209 women, 7462 men, 5272 girls and 5606 boys.

### Background characteristics of EHIS participants

The majority of the respondents were aged 15–59 in the 2015 EHIS. More than 6 in 10 women and men were living in rural areas. About half of women and men resided in Lower Egypt, and more than one-third lived in Upper Egypt. The educational level of the 2015 EHIS respondents varied considerably between women and men. Twenty-two percent of women aged 15–59 never attended school compared with eight percent of men. Among women, more than 40% had completed secondary school or higher, while more than 50% of men had completed secondary school or higher. Adult respondents in the EHIS were fairly evenly distributed across the wealth quintiles [15].

### Sampling technique of EHIS, 2015

The 2015 EHIS involved a systematic random selection of a subsample of 614 Shiakhas/villages out of the 884 shiakhas/villages that had been chosen as Primary Sampling Units in the 2014 EDHS. ﻿The household listings prepared during the 2014 EDHS were used to select the household sample for the EHIS. A total of 7,656 households were chosen from the EDHS listings in such manner the EHIS household sample was totally independent of the household sample selected for the EDHS. ﻿During the EHIS, usual household members and visitors who were present in the household during the night before the survey visit were identified and listed in the household questionnaire. All individuals 1–59 included in that list were eligible for the individual survey interview.

### Preparation of the study file

A special SPSS file has been prepared by El-Zanaty and Associates office in Egypt to include all girls aged (0–14) combined with their parents. The sample in this secondary analysis included 5,272 girls (1–14 years) obtained from EHIS 2015 together with their parents. The mothers and fathers included were 4,406 and 3,787 respectively.

### Study Variables

#### Outcome variables

##### Practicing FGM

This variable included current and intended circumcision status. The EHIS questionnaires asked the respondents about the circumcision status of their daughters and about the intention of mothers of uncut girls to perform circumcision in the future. To estimate the total girls who will be circumcised, a new variable was created by adding circumcised girls to those who were intended to be circumcised. Also, uncircumcised girls were added to those who were not intended to be circumcised in the future, to estimate the total uncircumcised girls.

##### FGM medicalization

Mothers of circumcised girls were asked about the perpetrator of FGM. We created a variable termed “FGM medicalization” denoting the performance of FGM by a health care provider (A physician or a nurse).

#### Independent variables

##### Sociodemographic characteristics of households

We used data about residence (urban/rural), region (Lower, Upper Egypt, urban and frontier governorates), wealth index and number of household members (Additional file 1).

##### Sociodemographic characteristics of the parents

We have also included the data of mothers’ and fathers’ age, education, occupation and religion.

##### Circumstances of circumcision

Age at circumcision, place and perpetuator of circumcision. Several indices have been created using different variables for both parents. For each of the following indices, separate indices for mothers and fathers were created.

##### Attitude towards FGM Index

Both mothers and fathers were asked about their beliefs regarding FGM. Seven questions were selected to capture the degree to which FGM attitudes may be entrenched in social, cultural and religious traditions; parents belief that FGM is religious, that FGM should continue, that other sex wants the practice to continue, husbands’ preference for circumcised wife, whether FGM affects child birth, prevents adultery or has severe consequences which can lead to death.

##### Decision-making power index

This index was created using five questions which asked about the decision maker in case of dealing with own earnings, spouse’s earnings, health care, household purchases and visits to family members. The responses to these questions were categorized into; a decision made by the husband, by the wife or jointly.

##### Attitude towards violence index

Parents’ attitude towards exposing wives to violence was assessed using questions about the justification of wife beating in case of; neglecting children, going out without husband’s permission, arguing with husband, refusing sex, and burning food.

### Statistical analysis

Descriptive statistics was performed in the form of frequencies, means and SD to describe the sample characteristics. The scores of the mothers’ and fathers’ attitude towards FGM were calculated by summation of the responses to the previously mentioned scale questions. The responses supporting the FGM practice were coded as “1”, and those against the practice were coded as “0”, with higher scores indicating the parent support of practicing FGM.

Another score was calculated for the mothers’ and fathers’ decision making also by summation of the participants’ response scores of the five questions assessing decision making. Responses were recoded as "0" for not participating entirely in decision making, "1" for taking the decision jointly and "2" for taking the decision alone, with higher scores indicating being more decisive. As most women were not working for cash, we didn’t use the question of the decision making regarding the use of the spouse’s earning in the fathers’ decision making index.

For the mothers' and fathers’ attitudes towards violence indices, responses supporting violence were recoded as "0" and responses against violence were recoded as "1", with higher scores indicating rejection of violence.

Cronbach’s alpha for all the created indices was more than 0.7 indicating good internal consistency.

Bivariate analysis using Chi-square test and t test were performed to identify the factors associated with the outcome variables of the study; circumcision status and medicalization of circumcision. The significant variables in the bivariate analysis were entered in a logistic regression model to identify the predictors of practicing FGM and medicalization. For all statistical tests used, statistical differences were considered significant when p-value was less than 0.05.

## Results

### Sociodemographic characteristics of the study girls

Table 1 shows the characteristics of the study girls. The mean age of circumcised and the uncircumcised girls was 11.1 ± 2.9 and 6.21 + 3.72, respectively. Girls residing in urban areas were less than those residing in rural areas, with the least frequency observed in frontier governorates. Nearly there was an even distribution of girls in the rich and poor wealth quintiles (41.4% of the girls were in the poor quintile and 42.2% of girls were in the rich quintile).

**Table 1:** Background characteristics of the study girls, EHIS 2015

Variables	Circumcised girls (n = 866)	Not circumcised (n = 4406)
	N (%)	N (%)
*Age*		
< 5	60 (2.7)	2139 (97.3)
5- < 11	209 (11.8)	1565 (88.2)
11–14	597 (46.7)	702 (54.0)
Mean ± SD	11.1 ± 2.9	6.21 + 3.72
*Place of residence*		
Urban	308 (13.4)	1982 (86.6)
Rural	558 (18.7)	2424 (81.3)
*Region*		
Urban governorates	61 (7.7)	730 (92.3)
Lower Egypt Urban	32 (5.7)	529 (94.3)
Lower Egypt Rural	148 (11.8)	1111 (88.2)
Upper Egypt Urban	170 (25.9)	487 (74.1)
Upper Egypt Rural	402 (24.8)	1216 (75.2)
Frontier governorates	53 (13.7)	333 (83.6)
*Wealth index*		
Poor	506 (23.1)	1680 (76.9)
Middle	119 (13.9)	737 (86.1)
Rich	241 (10.8)	1989 (89.0)
*Mothers' Age*		
< 30	119 (6.2)	1796 (93.8)
30- < 40	417 (19.1)	1767 (80.9)
≥ 40	298 (29.7)	706 (70.3)
Mean ± SD	37.8 ± 6.94	32.81 + 6.79
*Mothers' education*		
Below secondary	416 (24.1)	1313 (75.9)
Above secondary	418 (12.4)	2956 (87.6)
*Fathers' age*		
< 30	10 (2.6)	376 (97.4)
30- < 40	123 (8.2)	1370 (91.8)
40- < 45	472(24.7)	1436 (75.3)
Mean ± SD	44.0 ± 6.30	39.21 + 7.53
*Fathers' education*		
Below secondary	196 (19.7)	797 (80.3)
Above secondary	409 (14.6)	2385 (85.4)

Regarding their parents, just less than half of mothers were between the ages of 30–40 years and the majority has completed secondary education. Third of the fathers were between the ages of 30–40 years, and more than half have completed secondary school.

**Fig. 1:**
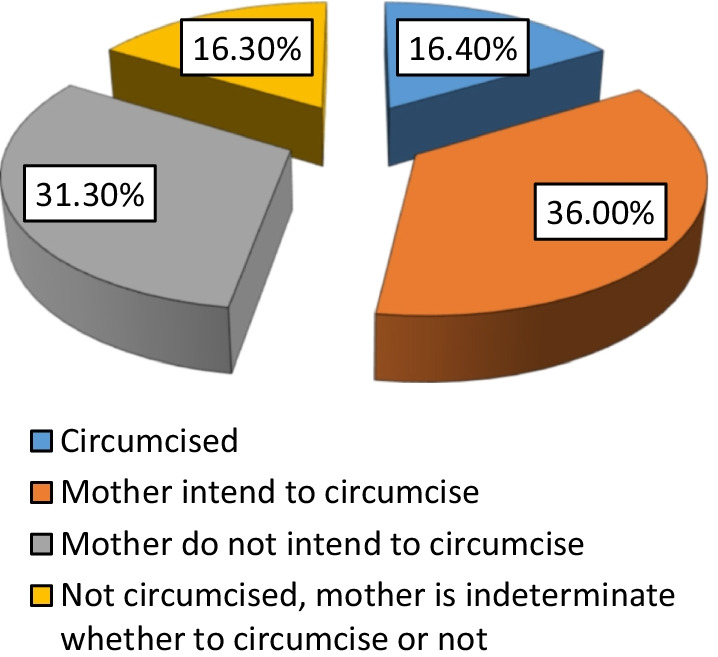
FGM status of the girls, EHIS 2015

Figure 1 shows that 16.4% of the study girls were circumcised, 36% of girls were expected to be circumcised as their mothers intended to. A considerable proportion of mothers (16.3%) were found to be indeterminate regarding their intention to circumcise their girls in the future, while only 31.3% of girls were not circumcised and their mothers didn’t intend to circumcise them in the future.

**Fig. 2:**
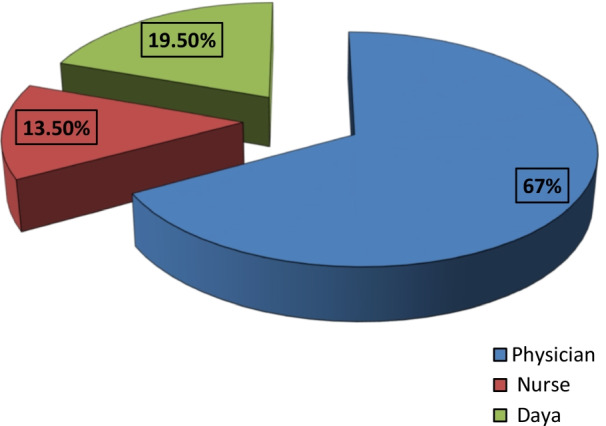
The perpetrator of FGM for the circumcised girls, EHIS, 2015

Figure 2 shows that more than two thirds (67%) of the circumcised girls were cut by a physician and 13.5% by nurses, while only one fifth of FGM (19.5%) were not performed by a health care specialist.

### Parents’ attitudes towards practicing FGM

Regarding the practice of circumcision, girls were classified into two groups: 1- Circumcised girls and girls intended to be circumcised 2- Not circumcised girls and girls not intended to be circumcised**.**

Table 2 shows that practicing FGM and the intention to circumcise girls were significantly associated with some mothers' and fathers’ beliefs. It was found that the majority of mothers and fathers who circumcised or intended to circumcise their daughters believed that FGM should continue (88.4% and 84.8%, respectively).They also believed that FGM is a religious obligation (72.3% of mothers and 73% of fathers). Some attitudes have also supported the males’ role in supporting the practice of FGM as most parents of circumcised girls believed that husbands would prefer the circumcised girls for marriage (81.1% and 82.5% of mothers and fathers respectively), and 78.1% of mothers believed that men want the practice to continue. It was also observed that parents of circumcised girls had less perception regarding the serious health complications of FGM and its effect on a difficult childbirth and believed more in the role of FGM preventing adultery than parents of uncircumcised girls. For all observed frequencies, there were statistically significant differences between mothers and fathers who have circumcised their girls and those intending to circumcise their girls and those who did not and were not intending to circumcise their girls (p =  < 0.001).

**Table 2:** Attitudes of mothers and fathers towards practicing FGM, EHIS 2015

	Mothers’ attitudes		Fathers’ attitudes	
	Circumcised & intended to be circumcised	Not circumcised & not indented to be circumcised	Circumcised & intended to be circumcised	Not circumcised & not intended to be circumcised
	No. (%)	No.(%)	No. (%)	No.(%)
*Believe FGM should continue*				
Yes	2358 (88.4)	266 (16.6)***	1653 (84.8)	392 (33.3)***
No	310 (11.6)	1336 (83.4)	297 (15.2)	784 (66.7)
*Believe FGM is required by religion*				
Yes	1931 (72.3)	293 (18.3)**	1423 (73.0)	352 (29.9)**
No	739 (27.7)	1309 (81.7)	527 (27.0)	824 (70.1)
*Believe the other sex wants FGM practice to continue*				
Yes	2082 (78.1)	328 (20.5)***	1460 (74.9)	304 (25.9)***
No	584 (21.9)	1274 (79.5)	489 (25.1)	872 (74.1)
*Believe husbands prefer a circumcised wife*				
Yes	2165 (81.1)	345 (21.5)***	1608 (82.5)	395 (33.6)***
No	505 (18.9)	1256 (78.5)	342 (17.5)	781 (66.4)
*Believe FGM makes childbirth difficult*				
Yes	220 (8.2)	189 (11.8)***	124 (6.4)	107 (9.1)**
No	2450 (91.8)	1412 (88.2)	1826 (93.6)	1068 (90.9)
*Believe FGM causes severe consequences that can lead to death*				
Yes	1255 (47.0)	1329 (83.0)***	791 (40.6)	820 (69.7)***
No	1413 (53.0)	272 (17.0)	1159 (59.4)	356 (30.3)
*Believe FGM prevents adultery*				
Yes	1810 (67.8)	264 (16.5)***	1421 (72.9)	346 (29.4)***
No	860 (32.2)	1337 (83.5)	529 (27.1)	830 (70.6)

^*^p < 0.05

^**^p < 0.01

^***^p < 0.001

### Predictors of practicing FGM and its medicalization

Bivariate analysis showed that circumcised girls and those intended to be circumcised were more likely to be residing in rural areas; 75.8% of girls in rural areas versus 45.4% of girls in urban areas, specifically in rural Upper Egypt (84.6%, p =  < 0.001), as compared to other regions in Egypt. Being circumcised and intended to be circumcised was also significantly associated with being in the poorest wealth quintile (77.8%, 71.5% and 43.2% in the poor, middle and rich wealth quintiles respectively, p =  < 0.001). Higher frequency of FGM was also observed among girls of below secondary educated mothers (74.8%), as compared to 55.7% of girls of above secondary educated mothers, p =  < 0.001 (Table 3).

**Table 3:** Factors affecting practice and medicalization of FGM

	Practicing FGM		Medicalization of FGM	
	Circumcised and intended to be circumcised	Not circumcised and Not intended to be circumcised	HCPs (Physician or Nurse)	Daya
	N (%)	N (%)	N (%)	N (%)
*Place of residence*				
Urban	868 (45.4)	1046 (54.6)***	261 (84.7)	47 (15.3)*
Rural	1897 (75.8)	604 (24.2)	435 (78.2)	121 (21.8)
*Region*				
Urban governorates	238 (36.5)	414 (63.5)***	51 (83.6)	10 (16.4)**
Lower Egypt Urban	152 (34.1)	294 (65.9)	30 (93.8)	2 (6.3)
Lower Egypt Rural	646 (67.4)	312 (32.6)	127 (86.4)	20 (13.6)
Upper Egypt Urban	361 (63.1)	211 (36.9)	144 (84.7)	26 (15.3)
Upper Egypt Rural	1225 (84.6)	223 (15.4)	303 (75.6)	98 (24.4)
Frontier governorates	143 (42.2)	196 (57.8)	41 (77.4)	12 (22.6)
*Wealth index*				
Poorest	842 (80.9)	199 (19.1)***	256 (80.0)	64 (20.0)***
Poorer	639 (74.1)	223 (25.9)	139 (75.1)	46 (24.9)
Middle	504 (71.5)	201 (28.5)	99 (83.9)	19 (16.1)
Richer	493 (57.5)	361 (42.3)	121 (80.1)	30 (19.9)
Richest	287 (30.1)	666 (69.9)	81 (90.0)	9 (10.0)
*Mothers education*				
Below secondary	1138 (74.8)	383 (25.2)***	308 (74.0)	108 (26.0)***
Above secondary	1532 (55.7)	1219 (44.3)	362 (86.8)	55 (13.2)
*Fathers education*				
Below secondary	579 (69.0)	260 (31.0)***	148 (75.5)	48 (24.5)***
Above secondary	1371 (59.9)	916(40.1)	356 (87.3)	52 (12.7)
*Parental perceptions*				
Mothers’ attitude supporting the practice of FGM	5.27 ± 1.69	2.04 ± 1.79***	4.91 ± 1.80	5.24 ± 1.84***
Mothers’ attitude against violence	4.00 ± 1.41	4.50 ± 1.02***	4.01 ± 1.42	3.53 ± 1.52
Mothers’ decision making index	3.79 ± 2.00	3.71 ± 1.81	3.88 ± 1.86	3.66 ± 1.89
Fathers’ attitude supporting the practice of FGM	4.92 ± 1.67	3.02 ± 1.86***	4.80 ± 1.67	4.76 ± 1.53***
Fathers attitude against violence	3.97 ± 1.45	4.37 ± 1.62***	4.09 ± 1.44	3.55 ± 1.49***
Fathers’ decision making index	6.02 ± 1.57	5.76 ± 1.65***	5.78 ± 1.56	5.90 ± 1.59

Significance: ***: *p* < .001, **: *p* < .010; *: *p* < 0.050

On the contrary, medicalization was found to be higher in urban areas (84.7% of circumcised girls in urban areas versus 78.2% in rural areas). Richer people were more likely to choose a health care provider for circumcision (83.8%, 83.9%, 78.2% of the rich, middle and poor wealth quintiles. Higher education of both mothers and fathers accounted for higher proportions of circumcision by health care providers (Table 3).

It was also found that mothers and fathers of circumcised girls and those intended to be circumcised had statistically significant higher scores of accepting FGM and lower scores of rejecting violence. However, fathers and mothers who have performed a medicalized FGM for their daughters had statistically significant higher scores of rejecting violence than those who have performed a daya-assisted FGM.

Table 4 shows the results of multivariable logistic regression analysis. Compared to frontier governorates, girls from rural Upper Egypt were more likely to be circumcised (odds ratio (OR) = 4.55, p < 0.001). Parents in the poorest wealth quintile were four times more likely to circumcise their girls, as compared to the richest (OR = 4.2, p < 0.001). Living in Rural Upper Egypt was also significantly associated with the FGM practice (OR = 4.55, p < 0.001). Mothers (OR = 1.97, p < 0.001) and fathers (OR = 1.27, p < 0.001) with higher scores of accepting FGM were also more likely to circumcise their girls. Moreover, fathers who were against exposing women to violence were more likely to leave daughters uncut (OR = 0.89, p < 0.05).

**Table 4:** Predictors of practicing and medicalization of FGM, EHIS, 2015

	Practicing FGM	Medicalization of FGM
	OR (95% confidence interval)	OR (95% confidence interval)
*Residence*		
Urban	Ref	Ref
Rural	2.65 (1.31–6.22)*	0.35 (0.02–4.33)
*Region*		
Frontier governorates	Ref	Ref
Urban governorates	0.58 (0.34–0.99)*	2.44 (0.72–8.31)
Lower Egypt Urban	0.45 (0.25–0.81)*	2.61 (0.67–10.01)
Lower Egypt Rural	1.96 (0.91–4.27)	1.23 (0.12–10.08)
Upper Egypt Urban	1.49 (0.88–2.52)	2.08 (0.72–6.05)
Upper Egypt Rural	4.55 (2.12–9.77)***	0.68 (0.07–6.67)
*Wealth Index*		
Richest	Ref	Ref
Poorest	4.20 (2.46–7.16)***	0.34 (0.13–0.83)**
Poorer	2.24 (1.29–3.88)**	0.46 (0.18–1.16)
Middle	2.85 (1.67–4.86)***	0.42 (0.15–1.11)
Richer	1.94 (1.33–2.81)***	1.07 (0.61–1.89)
*Mothers; education*		
Secondary and above	Ref	Ref
Below secondary	1.00 (0.74–1.35)	0.69 (0.43–1.10)
*Fathers’ education*		
Secondary and above	Ref	Ref
Below secondary	0.97 (0.71–1.33)	0.58 (0.34–0.98)*
*Mothers attitude supporting the practice of FGM*	1.97 (1.85–2.11)***	0.97 (0.86–1.09)
*Fathers attitude supporting the practice of FGM*	1.27 (1.20–1.35)***	1.10 (0.97–1.25)
*Fathers’ decision making*	0.97 (0.89–1.04)	
*Mothers’ attitude against violence*	0.93 (0.84–1.05)	
*Fathers attitude against violence*	0.89 (0.80–0.89)*	1.25 (1.08–1.45)**

Significance: ***: *p* < 0 .001, **: *p* < 0.01; *: *p* < 0.05

Regarding the predictors of medicalization, parents in the poorest quintile were less likely to circumcise their daughters by a health care provider (OR = 0.34, p < 0.01). As compared to having secondary education or higher, parents who had a below secondary education were less likely to perform a medicalized FGM (OR = 0.58, p < 0.05). Moreover, fathers who had an attitude rejecting violence against women were more likely to circumcise their girls by health care providers (OR = 1.25, p < 0.01).

## Discussion

The practice of FGM and its medicalization is still prevalent in many developing countries, with Egypt having the highest observed rates [16]. Secondary analysis of the EHIS data has supported this high rate of FGM medicalization observed in other studies [9, 17, 18], as it showed that 67% of the circumcised girls were circumcised by a physician and 13.5% by nurses.

Studying the parents’ attitudes towards FGM in the EHIS has revealed, despite all the national efforts combating FGM, an unexpected continuous support of the practice by both mothers and fathers, which was associated significantly with the actual performance and the intention to perform this practice for their daughters. It was found that about 62% and 65% of mothers and fathers who have participated in EHIS believed that FGM should continue. Several other studies in Egypt have also shown the continued community support for the practice of FGM whoever the perpetrator [5, 19–21]. A variety of social and cultural reasons for supporting FGM are reported, including female cleanliness, cultural identity, protection of virginity, prevention of immorality, better marriage prospects, greater pleasure for the husband and improvement of fertility [4, 22].

The demand for medicalized FGM appears to be driven by a number of factors. First, heightened concerns over potential health complications may have motivated parents to seek medicalized cutting, assuming it has less complications Moreover, mothers choose physicians for performing FGM because they are trustworthy, use sterile equipment and cut less amount of tissue [23–25]. Medicalization could have been driven also by policies restricting traditional practitioners, but allowing health professionals to perform FGM for harm reduction. In 1994, the Egyptian Minister of Health stated that physicians could perform FGM on girls in designated facilities at fixed times and prices, claiming that medicalization of the practice would reduce complications and eventually end the practice [26, 27]. However, this was unintentionally associated with legitimizing the practice and creating an impression that the procedure may be performed safety, undermining all efforts for abandonment of FGM and resulting in increased medicalization rates [27]. As a result, this policy was reversed to banning FGM/C in both state and private hospitals in 2008. In 2016, the law banning medical professionals from performing FGM in either state or privately-run clinics was amended to raise the maximum sentence from 3 to 15 years in prison.

This secondary analysis of EHIS data found a significant association between poverty, low education levels and practicing FGM. This could be attributed to the acceptance of FGM as an entrenched tradition and social norm in the poor communities where illiteracy prevails [28, 29]. Furthermore, the poor and less educated are ignorant about the negative consequences of FGM and don't consider women's sexual rights [5, 29]. These findings were consistent with other studies [17, 30, 31] as wealth is usually connected with other social parameters (e.g., place of residence and/or household level of education). Affluent women have strong decision-making power on harmful traditional practices like FGM on themselves and their daughters because of their wealth status [32]. Moreover, the association between FGM practice and level of education indicates that educationally empowered women are more likely to turn down any societal pressure to circumcise their daughters. The Educational status is also a proxy of the family background, with more openness about criticisms against this practice and a better awareness of its adverse effects [31].

EHIS data has shown that despite the observed higher rates of FGM among the poor, medicalization is more observed among the Rich. This finding was supported by SYPE which showed a higher frequency of having FGM at a private clinic (60.5%) when belonging to the highest wealth quintile, as compared to the poor quintile (19.1%). This could be attributed to the inability of the poor to afford the expenses of the medical procedure, in addition to the attempt of the rich parents to reduce harm by performing the practice under medical observation, while satisfying the community directed pressure of maintaining the practice [18].

In the same context, it was found that higher rates of FGM were observed in rural Upper Egypt, while higher rates of medicalization were found in urban Lower Egypt. This can be explained by the same conceptual framework associating medicalization with higher levels of education and economic sufficiency in urban areas of Lower Egypt as compared to poverty and ignorance in rural Upper Egypt. Moreover, the conservative nature of the rural community and the limited gender roles of girls in absence of education and employment have restricted the social position of women in getting married and avoiding adultery. In these communities, with women having no sources of empowerment, parents believe that they safeguard their daughters’ social position and marriageability through FGM [33]. Physicians support of the practice, more observed in the rural areas, should not be overlooked, as it was found in a previous study conducted in Egypt, that physicians may support the hesitant mothers for performing FGM in the poor rural communities either for financial incentives or for complying to the norms of the community where they work [5].

This secondary analysis has also highlighted the religious misconceptions of parents who have circumcised their daughters, where about 52% and 56% of mothers and fathers believed that FGM is a religious obligation by Islam. The FGM related religious beliefs were also evident in other studies [34, 35]. This finding highlights the importance of partnership between religious leaders and community groups to create awareness and openness for discussion, and correcting the religious misconceptions to help changing attitudes and contribute to abandonment of the practice of FGM [4].

As we hypothesized the important role of males in the practice continuation, we have examined the variable related to fathers’ attitudes about FGM practice, rejecting violence and their participation in decision making. Our hypothesis was in agreement with the observed parents’ beliefs related to male factors, such as the preference of a circumcised wives by husbands (59% of mothers and 64% of fathers) and the perception of women that the males wants the practice to continue (56% of mothers). This finding was consistent by other studies in Egypt [36–38]. Although lack of sexual response is inconvenient and disturbing to the husband, the practice is still deemed important to ensure marriage fidelity and retain the husband’s feeling of security [39]. Fathers’ role was also evident by the significant association between their supportive attitudes towards FGM and their actual and intended practice of circumcising their daughters. Some previous studies have also documented the decision making role of fathers in supporting the continuation of FGM practice [36, 38].

This study has also found significant associations between the fathers’ rejection of violence against women and their decision of circumcising their girls by a health care provider, and lower rates of practicing circumcision. Fathers rejecting violence may think of medicalization as a protection of their girls from the traumatic procedure of the extensive cutting performed by traditional practitioner without using any form of anaethesia [6]. However, it is always painful, traumatic with a series of short- and long-term consequences and violating a series of human rights principles, including the principles of equality and non-discrimination on the basis of sex.

It is to be mentioned that males are not only fathers but also husbands, community and religious leaders, which would definitely have a major role in supporting the continuation or abandonment of FGM practice [39]. In the absence of the active husbands’ support and other influential male leaders in the community, women who decide not to circumcise their daughters would face not only peer and community pressure, but also feelings of helplessness [40].

## Strengths and limitations

EHIS, 2015 included a nationally representative data from both men and women in Egypt, allowing the study of the attitudes of both parents towards FGM and its medicalization. However, we had some limitations, as the cross sectional nature of the study hindered the establishment of temporal associations, and did not allow us to know whether the intention of practicing FGM has been translated into actual practice.

## Implications

Investigating both parents’ attitudes toward FGM and its medicalization would guide government programming decisions to develop policies and strategies targeting both parents, not only mothers. Men must be targeted in FGM programs, as their views on FGM must be understood and integrated in efforts to address abandonment. A comprehensive approach to stop FGM is needed including all stakeholders, political, religious, cultural leadership and all members of social networks, which would help in mitigating social sanctions and stigma from failing to conform. More attention should be paid to motivating the role of girls’ education and employment, and raising the community awareness about rejecting violence against women and gender equity in sexual rights. We also recommend strengthening the facility level supervision mechanisms of Ministry of Health to stop health care providers from performing FGM, with activating laws and penalties on providers and families practicing FGM.

## Supplementary Information

**Additional file 1.** SPSS data file of EHIS, 2015, prepared by El-Zanaty and Associates office in Egypt. The file includes all girls aged (0-14) combined with their parents. The sample in this secondary analysis included 5272 girls. The mothers and fathers included were 4406 and 3787 respectively.

## Data Availability

All data generated or analysed during this study are included in this published article and its supplementary information files.

## References

[1] Leye E, Van Eekert N, Shamu S, Esho T, Barrett H (2019). Debating medicalization of female genital mutilation/cutting (FGM/C): learning from (policy) experiences across countries. Reprod Health.

[2] Serour GI (2013). Medicalization of female genital mutilation/cutting. Afr J Urol.

[3] Brief on the medicalization of female genital mutilation | United Nations Population Fund [Internet]. [cited 2021 Nov 7]. https://www.unfpa.org/resources/brief-medicalization-female-genital-mutilation

[4] McCauley M, Van Den Broek N (2019). Challenges in the eradication of female genital mutilation/cutting. Int Health.

[5] El-Gibaly O, Aziz M, Abou HS (2019). Health care providers’ and mothers’ perceptions about the medicalization of female genital mutilation or cutting in Egypt: a cross-sectional qualitative study. BMC Int Health Hum Rights.

[6] Muteshi JK, Miller S, Belizán JM (2016). The ongoing violence against women: female genital mutilation/cutting. Reprod Health.

[7] Wilson Ann-Marie. Country Profile: FGM in Egypt. 2017;52

[8] Johansen REB, Ahmed SAE (2021). Negotiating female genital cutting in a transnational context. Qual Health Res.

[9] Van Rossem R, Meekers D, Gage AJ (2015). Women’s position and attitudes towards female genital mutilation in Egypt: a secondary analysis of the Egypt demographic and health surveys, 1995–2014. BMC Public Health.

[10] Zanty F. Dhs 2014. 2014;1–246. http://dhsprogram.com/pubs/pdf/FR302/FR302.pdf

[11] Egypt, Arab Rep. - Demographic and Health Survey 2008 [Internet]. [cited 2021 Nov 7]. https://microdata.worldbank.org/index.php/catalog/1376

[12] Pearce AJ, Bewley S (2014). Medicalization of female genital mutilation Harm reduction or unethical?. Obstet Gynaecol Reprod Med.

[13] Alradie-Mohamed A, Kabir R, Arafat SMY (2020). Decision-making process in female genital mutilation: a systematic review. Int J Environ Res Public Health.

[14] Eldin A, Babiker S, Sabahelzain M, Eltayeb M. FGM/C decision-making process and the role of gender power relations in Sudan. Reprod Health. 2018.

[15] Egypt Health Issues Survey | UNICEF Egypt [Internet]. [cited 2021 Nov 6]. https://www.unicef.org/egypt/reports/egypt-health-issues-survey

[16] Female genital mutilation [Internet]. [cited 2021 Nov 6]. https://www.who.int/news-room/fact-sheets/detail/female-genital-mutilation

[17] Survey of Young People in Egypt | Population Council [Internet]. [cited 2021 Nov 7]. https://www.popcouncil.org/research/survey-of-young-people-in-egypt

[18] Ghattass S, Abdel-Tawab NG, Hussein SA. Ending the medicalization of female genital mutilation/cutting in Ending the medicalization of female genital mutilation/cutting in Egypt Egypt: Policy brief. Reprod Heal Soc Behav Sci Res. 2016. https://knowledgecommons.popcouncil.org/departments_sbsr-rh

[19] Dalal K, Kalmatayeva Z, Mandal S, Ussatayeva G, Lee MS, Biswas A (2018). Adolescent girls’ attitudes toward female genital mutilation: a study in seven African countries. F1000Research.

[20] Alkhalaileh D, Hayford SR, Norris AH, Gallo MF (2018). Prevalence and attitudes on female genital mutilation/cutting in Egypt since criminalisation in 2008. Cult Health Sex.

[21] Abdou MSM, Wahdan IMH, El-Nimr NA (2020). Prevalence of female genital mutilation, and women’s knowledge, attitude, and intention to practice in Egypt: a nationwide survey. J High Inst Public Heal.

[22] WADI. Final Report. Stop FGM Middle East. 2014;1–18

[23] Kimani S, Shell-Duncan B (2018). Medicalized female genital mutilation/cutting: contentious practices and persistent debates. Curr Sex Heal Rep.

[24] Doucet MH, Pallitto C, Groleau D (2017). Understanding the motivations of health-care providers in performing female genital mutilation: an integrative review of the literature. Reprod Health.

[25] Modrek S, Sieverding M (2016). Mother, daughter, doctor: Medical professionals and mothers’ decision making about female genital cutting in Egypt. Int Perspect Sex Reprod Health.

[26] Shell-Duncan B (2001). The medicalization of female “circumcision”: Harm reduction or promotion of a dangerous practice?. Soc Sci Med.

[27] Edy SM (2019). Preventing health-care providers from performing female genital mutilation. J Chem Inf Model.

[28] In CN, Wild T, Diagnose HT, Norms CS. Dynamics of a Social Norm: 2016

[29] Barrett HR, Brown K, Alhassan Y, Leye E (2020). Transforming social norms to end FGM in the EU: an evaluation of the REPLACE approach. Reprod Health.

[30] Setegn T, Lakew Y, Deribe K (2016). Geographic variation and factors associated with female genital mutilation among reproductive age women in Ethiopia: a national population based survey. PLoS ONE.

[31] Ahinkorah BO, Hagan JE, Ameyaw EK, Seidu AA, Budu E, Sambah F (2020). Socio-economic and demographic determinants of female genital mutilation in sub-Saharan Africa: analysis of data from demographic and health surveys. Reprod Health.

[32] Rose L (2017). Women’s healthcare decision-making autonomy by wealth quintile from demographic and health surveys (DHS) in Sub-Saharan African Countries. Int J Womens Heal Well.

[33] Child marriage and FGM/C: What you need to know - Girls Not Brides [Internet]. [cited 2021 Nov 8]. https://www.girlsnotbrides.org/articles/child-marriage-fgmc-need-know/

[34] Hayford SR, Trinitapoli J (2011). Religious differences in female genital cutting: a case study from Burkina Faso. J Sci Study Relig.

[35] Religious Perceptions and Attitudes of Men towards Discontinuation of Female Genital Cutting in Nigeria on JSTOR [Internet]. [cited 2021 Nov 9]. https://www.jstor.org/stable/26493897 10.29063/ajrh2018/v22i1.2 29777639

[36] Shaheen H, Kasemy Z, Salah EF (2017). The current situation regarding awareness about female genital mutilation among men working in schools of Benha City, Qaluobia Governorate. Menoufia Med J.

[37] Abdelshahid A, Campbell C (2015). ‘Should I Circumcise My Daughter?’ Exploring diversity and ambivalence in Egyptian parents’ social representations of female circumcision. J Community Appl Soc Psychol.

[38] Kaplan A, Cham B, Njie LA, Seixas A, Blanco S, Utzet M (2013). Female genital mutilation/cutting: the secret world of women as seen by men. Obstet Gynecol Int.

[39] Varol N, Turkmani S, Black K, Hall J, Dawson A (2015). The role of men in abandonment of female genital mutilation: a systematic review. BMC Public Health.

[40] Al-Khulaidi GA, Nakamura K, Seino K, Kizuki M (2013). Decline of supportive attitudes among husbands toward female genital mutilation and its association to those practices in Yemen. PLoS ONE.

